# Staged Bilateral Microvascular Decompression for Simultaneous Bilateral Trigeminal Neuralgia Associated With Dolichoectatic Neurovascular Conflict: A Case Report and Literature Review

**DOI:** 10.7759/cureus.104303

**Published:** 2026-02-26

**Authors:** Jesús Oswaldo Díaz Lagunas, Tania Jimenez Molina, Rogelio Revuelta, Alejandro Becerril-Mejía

**Affiliations:** 1 Functional Neurosurgery, National Institute of Neurology and Neurosurgery "Manuel Velasco Suárez", Mexico City, MEX; 2 Functional Neurosurgery, National Autonomous University of Mexico, Mexico City, MEX; 3 Neurosurgery, National Institute of Neurology and Neurosurgery "Manuel Velasco Suárez", Mexico City, MEX

**Keywords:** dolichoectasia, functional neurosurgery, microvascular decompression (mvd), neurovascular conflict, simultaneous bilateral trigeminal neuralgia

## Abstract

Trigeminal neuralgia is most commonly unilateral and caused by neurovascular compression at the trigeminal root entry zone. Bilateral trigeminal neuralgia is rare, and simultaneous bilateral involvement is exceptionally uncommon, particularly when associated with dolichoectatic arterial conflict.

A 72-year-old man presented with a five-year history of medically refractory simultaneous bilateral trigeminal neuralgia. Right-sided pain involved the V1, V2, and V3 divisions and consisted of high-intensity, electric shock-like paroxysms occurring more than seven times daily, followed by continuous pain lasting up to three hours. Pain severity was rated as Barrow Neurological Institute score 5 and Numerical Rating Scale 8 out of 10. Left-sided pain developed one year later, affected the V3 division, and manifested as more than five daily paroxysms of severe intensity (Barrow score 4; pain scale 10 out of 10), markedly limiting mastication and daily activities. Carbamazepine 600 mg/day was ineffective and poorly tolerated, and symptom progression led to depression, anxiety, avoidance behaviors, and suicidal ideation, with a Karnofsky Performance Status of 60. High-resolution MRI with contrast-enhanced T2-weighted fast imaging employing steady-state acquisition (FIESTA) sequences demonstrated bilateral neurovascular compression with a dolichoectatic superior cerebellar artery (SCA) and multiple neurovascular conflicts. The patient underwent staged bilateral microvascular decompression via mini-retrosigmoid approaches during a single hospitalization, with the right side operated on first due to more disabling continuous pain. Both procedures achieved immediate, complete, and complication-free pain relief, allowing withdrawal of medication and functional recovery (Karnofsky Performance Status of 90).

Simultaneous bilateral trigeminal neuralgia with dolichoectatic neurovascular conflict poses a significant diagnostic and therapeutic challenge. This case supports staged bilateral microvascular decompression within a single admission as a safe and effective option for carefully selected patients with clear bilateral neurovascular compression.

## Introduction

The third edition of the International Classification of Headache Disorders (ICHD-3) defines trigeminal neuralgia (TN) as a facial pain syndrome characterized by recurrent, unilateral, brief, electric shock-like paroxysms confined to one or more divisions of the trigeminal nerve, typically triggered by innocuous stimuli such as light touch, talking, or chewing [1]. TN has an estimated prevalence of approximately 0.03% and an annual incidence of about 4.3 cases per 100,000 individuals, with increasing frequency in older adults [1,2]. Classical TN is most commonly attributed to neurovascular compression at the trigeminal root entry zone, whereas secondary TN is associated with structural lesions such as tumors, demyelinating plaques, or other pathologies affecting the nerve or brainstem [2].

Bilateral trigeminal neuralgia (BTN) is uncommon and falls outside the strict ICHD-3 criteria, which emphasize unilateral pain [1]. A recent systematic review reported an incidence of BTN of about 1.6% among patients with TN, most frequently with sequential onset, in which contralateral symptoms appear years after the initial side is affected [3]. Simultaneous BTN, with near-concurrent onset of pain on both sides of the face, is exceedingly rare, and its true incidence remains undefined [3,4]. Etiologic factors proposed for BTN include reduced posterior fossa volume, skull base abnormalities, genetic susceptibility, and systemic autoimmune or demyelinating diseases, although most cases are still considered sporadic [2,5-11].

Patients with TN who respond inadequately to pharmacological therapy and have features suggestive of systemic disease should be evaluated for underlying conditions such as multiple sclerosis, systemic lupus erythematosus, systemic sclerosis, polymyositis/dermatomyositis, primary Sjögren’s syndrome, and rheumatoid arthritis [6,8]. Multiple sclerosis is particularly relevant in BTN, as demyelinating plaques involving the trigeminal root can directly cause facial pain, and bilateral involvement is more frequently observed in this context [5,8]. In contrast, classical BTN, due to bilateral neurovascular conflict, requires careful neuroimaging assessment to demonstrate clinically significant compression on both sides and to exclude secondary etiologies.

Several interventional treatments are available for medically refractory TN, including microvascular decompression (MVD), percutaneous rhizotomy techniques, and stereotactic radiosurgery [2,12-14]. MVD is considered the preferred option for classical TN because it addresses the underlying neurovascular conflict and offers the most durable long-term pain relief with a relatively low complication rate [2,12]. However, the optimal management of BTN (particularly simultaneous bilateral disease associated with dolichoectatic vessels) remains a therapeutic challenge, and the role of bilateral MVD, whether staged or performed consecutively, is still being defined in the literature [3,4,8,11,15,16].

We report a rare case of simultaneous BTN associated with bilateral dolichoectatic neurovascular conflict, successfully treated with staged bilateral MVD during a single hospitalization, and provide a focused review of the literature on bilateral MVD for BTN.

## Case presentation

In March 2023, a 72-year-old man presented with simultaneous BTN. Symptoms began five years earlier with right-sided hemifacial pain involving the V1, V2, and V3 divisions of the trigeminal nerve, characterized by high-intensity, electric shock-like paroxysms (Barrow Neurological Institute [BNI] score 4; Numerical Rating Scale [NRS] 10/10) [17] occurring more than seven times daily, either spontaneously or triggered by facial contact, toothbrushing, or eating. One year later, he developed left-sided hemifacial pain confined to the V3 distribution, with more than five daily high-intensity paroxysms (BNI 4; NRS 10/10), which impeded eating and often caused uncertainty about the side of origin.

Before surgery, right-sided TN remained intense and involved the V1-V3 divisions, but had evolved to include a lower-intensity continuous pain lasting up to two hours after each paroxysm (BNI 5; NRS 8/10). Left TN involved V3 and continued to be paroxysmal (BNI 4; NRS 10/10). Pain control was inadequate despite carbamazepine 600 mg/day, which caused dizziness and somnolence. Symptom progression was accompanied by depression, anxiety, fear of driving and sexual activity, and emerging suicidal ideation. The Karnofsky Performance Status (KPS) [18] was 60, indicating an inability to work with preservation of most self-care activities.

**Figure 1:**
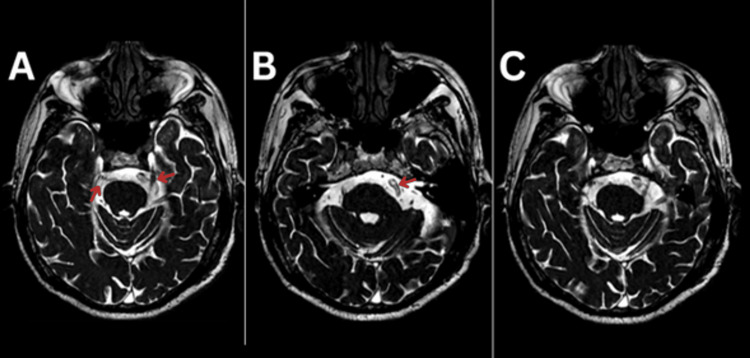
Preoperative MRI of contrast-enhanced T2-weighted fast imaging employing steady-state acquisition (FIESTA) sequence A) Bilateral neurovascular conflict; B) Visible dolichoectatic artery; C) Multiple neurovascular conflicts.

A comprehensive diagnostic workup was performed. Neurological examination did not reveal facial sensory loss, motor deficits, or other cranial nerve abnormalities. Laboratory studies and systemic evaluation did not show evidence of demyelinating, infectious, or systemic autoimmune disease. Brain MRI with high-resolution contrast-enhanced T2-weighted fast imaging employing steady-state acquisition (FIESTA) sequences demonstrated bilateral neurovascular contact at the trigeminal root entry zones with arterial dolichoectasia and nerve displacement, consistent with clinically significant bilateral neurovascular conflict (Figure 1). No demyelinating plaques, mass lesions, or other structural abnormalities were identified.

Because the right-sided pain was continuous and more disabling, right-sided MVD via a mini-retrosigmoid approach was performed first. The procedure resulted in immediate and complete pain relief on the right side (BNI 1; NRS 0) without complications. A four-day interval was chosen to allow careful monitoring for early postoperative complications after the first procedure, including cerebrospinal fluid leak, cerebellar edema, and new cranial nerve deficits, while avoiding a second hospital admission. Once the patient remained clinically stable with complete right-sided pain relief, left-sided MVD was performed using the same approach, again producing immediate and complete pain resolution (BNI 1; NRS 0) and no new neurological deficits. Postoperatively, the patient discontinued all antineuralgic medications and reported significant emotional and functional improvement, with the KPS improving to 90. At one, three, six, and 12 months of follow-up, he remained completely pain-free on both sides (BNI 1; NRS 0), without recurrence of paroxysmal or continuous pain and without new sensory deficits, hearing loss, or vestibular symptoms.

Surgical technique

The mini-retrosigmoid approach was used to access the cerebellopontine angle and the trigeminal root entry zone. Under general anesthesia, the patient was positioned in the lateral decubitus (Park-Bench) position, with the head secured in a three-pin Mayfield holder. The head was rotated approximately 10 degrees toward the contralateral side and slightly extended in the coronal plane to align the superior sagittal sinus parallel to the floor, thereby positioning the trigeminal nerve superior to cranial nerves VII and VIII. A vertical skin incision was made 1 cm above and 3 cm below the asterion. A burr hole was placed at the inferomedial junction of the transverse and sigmoid sinuses and enlarged with a Kerrison rongeur to create a craniectomy of approximately 25 mm in diameter. The dura was opened in a superiorly based C-shaped fashion. After the surgical microscope was introduced, the cerebellum was protected with oxidized cellulose, and gradual medial relaxation was achieved using sequentially placed cottonoid patties and gentle dynamic retraction, keeping retraction to a minimum under direct visualization of cranial nerves VII and VIII to preserve a safe anatomical corridor and reduce the risk of vestibulocochlear injury. Intraoperative neuromonitoring of cranial nerve VIII with brainstem auditory evoked potentials was not available for this case; therefore, additional safety measures included extensive cerebrospinal fluid drainage to minimize cerebellar retraction, strict avoidance of bipolar coagulation and excessive thermal energy near the internal auditory canal and cerebellopontine angle, and reliance on continuous microscopic inspection of cranial nerves VII and VIII throughout the procedure. As part of our institutional protocol, patients undergoing posterior fossa surgery with potential risk to cranial nerve VIII are evaluated preoperatively by the neuro-otology service, including baseline pure-tone audiometry, and are reassessed 24-48 hours after surgery and at three- and six-month follow-up; in this patient, no new hearing loss or vestibular symptoms were detected at any time point.

**Figure 2:**
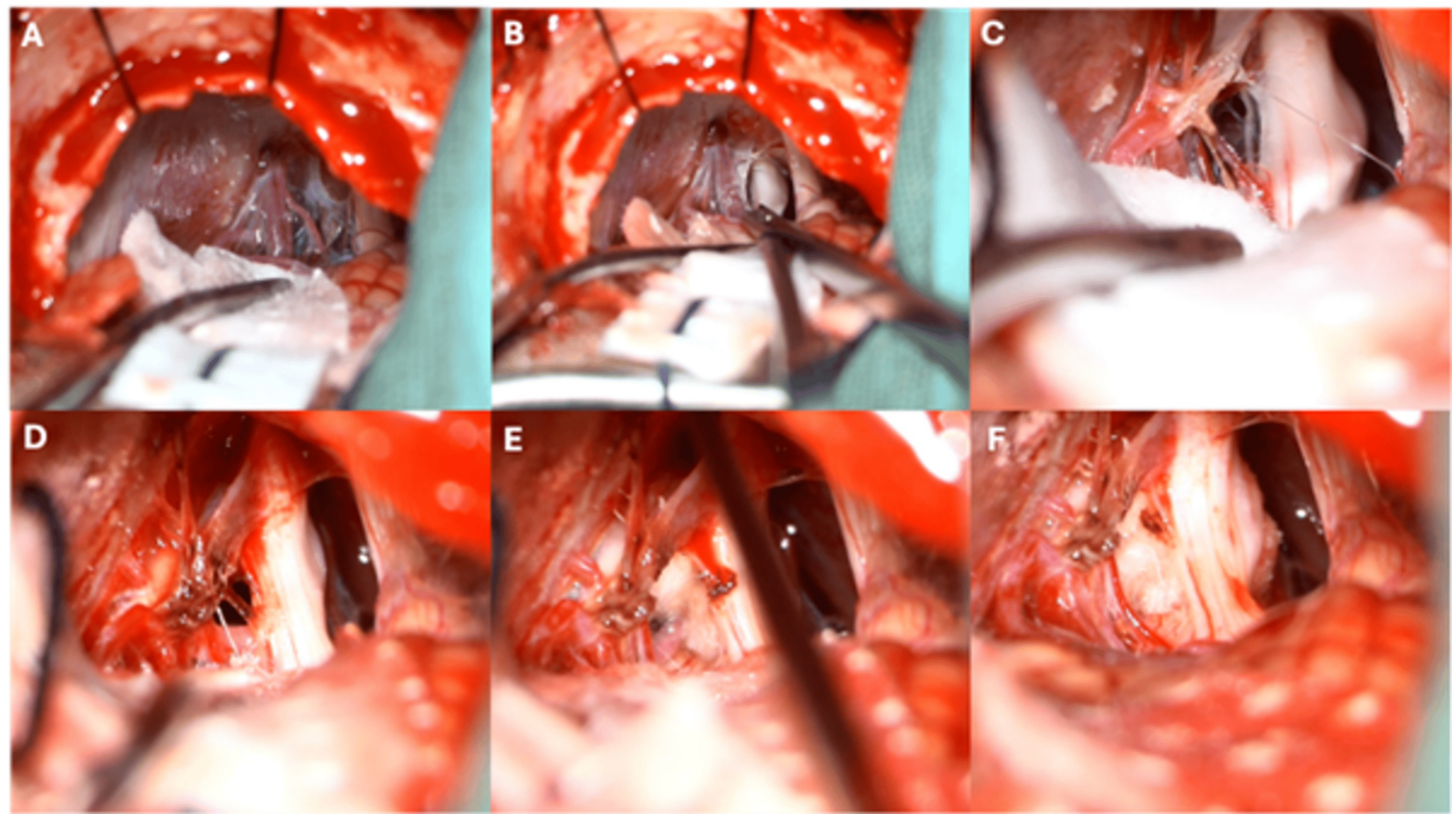
Intraoperative images through a right asterional approach. A) After drainage of the pontocerebellar cistern, the neurovascular complex is visualised covered by arachnoid. B) Sharp arachnoid dissection is performed to expose the trigeminal nerve. C) Anteroposterior vascular contact from the superior cerebellar artery (SCA) producing indentation of the trigeminal nerve. D) The SCA and trigeminal nerve are fully exposed; the latter followed to its entry zone into the pons. E) Placement of Teflon pledgets to achieve separation between the artery and the nerve. F) Final operative view showing cottonoids interposed between the SCA and the right trigeminal nerve, with a smooth and restored nerve surface and no residual vascular compression.

**Figure 3:**
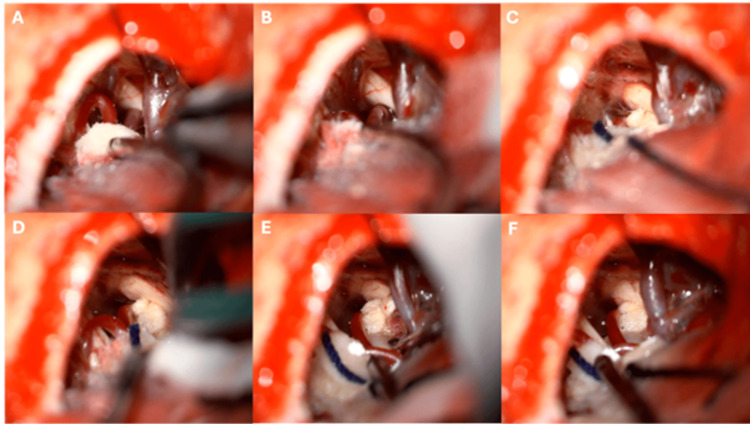
Intraoperative images through a left asterional approach A) Following drainage of the pontocerebellar cistern, the neurovascular complex is visualised, with the superior petrosal vein identified in the upper portion of the field. B) Dorsomedial vascular contact from the superior cerebellar artery (SCA) producing deformation of the trigeminal nerve. C) Placement of the first Teflon pledget to separate the artery from the nerve. D) Placement of a second Teflon pledget, achieving further decompression of the trigeminal nerve from the SCA. E) Placement of a third Teflon pledget along the posterior aspect of the trigeminal nerve, providing additional separation from both the SCA and the superior petrosal vein. F) Final operative view demonstrating complete microvascular decompression, with cottonoids interposed between the SCA, the superior petrosal vein, and the left trigeminal nerve.

Under high microscopic magnification, cerebrospinal fluid was released from the cisterna magna and the cerebellopontine angle cistern to further relax the cerebellum. The arachnoid membranes over the trigeminal nerve and the petrosal venous complex were carefully opened to allow thorough inspection. The superior petrosal vein was preserved when possible; when it became taut and impeded adequate visualization, it was coagulated and divided. The trigeminal nerve was examined circumferentially at the root entry zone. On each side, the offending vessel was identified as a dolichoectatic superior cerebellar artery (SCA) loop compressing and displacing the trigeminal root. Decompression was performed by mobilizing the vessel away from the nerve and interposing multiple small Teflon pledgets to create a stable “bridge” between the artery and the trigeminal nerve, taking care to avoid excessive packing or kinking of the vessel (Figures 2, 3).

Dural closure was performed hermetically using 4-0 Prolene. Larger dural defects were reconstructed with autologous fascia or muscle. Bone wax was applied to the cranial margins, the bone flap was repositioned, and the wound was closed in anatomical layers.

## Discussion

TN is a multifactorial disorder in which neurovascular compression at the trigeminal root entry zone, anatomical variants, genetic susceptibility, and central neurophysiological mechanisms interact to produce neuronal hyperexcitability and ectopic discharges [2,5,19]. Classical TN is usually unilateral and attributed to a focal neurovascular conflict, whereas BTN is uncommon and often associated with secondary causes such as multiple sclerosis, tumors, or systemic autoimmune disease [2,5,6,8]. A recent systematic review estimates that BTN occurs in approximately 1.6% of patients with TN, most often with sequential rather than simultaneous onset, and the incidence of truly simultaneous BTN remains undefined [3,4].

Bilateral trigeminal neuralgia: epidemiology and etiologic considerations

In this context, distinguishing classical BTN due to bilateral neurovascular conflict from alternative etiologies is essential, as it directly influences management [2,5]. Patients with TN who respond inadequately to pharmacological therapy and exhibit autoimmune risk factors should be evaluated for underlying systemic disease, including multiple sclerosis, systemic lupus erythematosus, systemic sclerosis, polymyositis/dermatomyositis, primary Sjögren's syndrome, and rheumatoid arthritis [6,8]. Multiple sclerosis is present in approximately 4-10% of BTN cases, and the prevalence of TN among individuals with multiple sclerosis is estimated at 3.4% [6]. Demyelinating plaques involving the trigeminal root can directly produce TN, and the presence of BTN in a relatively young patient should raise suspicion for demyelinating disease [5,6,8].

Reduced posterior fossa volume is a major proposed risk factor for BTN, exemplified by conditions such as Dandy-Walker malformation, where restricted space increases the likelihood of neurovascular compression [7]. Skull base anatomical abnormalities and candidate genes involving ion channels, myelin, and connective tissue have also been implicated in predisposition to BTN, although most cases remain sporadic [5,7,9]. The higher prevalence of TN in women may relate to smaller posterior fossa volume and to sex-related differences in neuroinflammation, hormonal modulation, and pain-processing pathways [5,10]. Additionally, familial TN has been reported at a higher rate in BTN patients compared to those with unilateral disease, suggesting a potential genetic predisposition to symptomatic neurovascular compression [11].

Diagnostic approach and neuroimaging

Neurovascular contact is frequent in both symptomatic and asymptomatic individuals and therefore does not by itself establish causality [20]. Clinically significant compression is suggested by pulsatile arterial contact, involvement of the root entry zone, nerve displacement or indentation, and age-related trigeminal vulnerability, all of which increase the likelihood that the contact is pathophysiologically relevant [5,20]. In our patient, high-resolution MRI with contrast-enhanced T2-weighted FIESTA imaging showed bilateral dolichoectatic arterial compression with evident displacement of the trigeminal roots, strongly supporting a causal neurovascular conflict on both sides. The absence of demyelinating plaques or mass lesions further argued against multiple sclerosis or tumor as primary etiologies. High-resolution 3D T2-weighted, 3D time-of-flight, MR angiography, and contrast-enhanced 3D T1-weighted sequences are essential for delineating neurovascular contact and root compression [2].

Another relevant feature in this case was the coexistence of paroxysmal and continuous pain. Prospective studies indicate that continuous background pain often precedes or accompanies paroxysmal attacks, is associated with sensory abnormalities and altered brainstem reflexes, and may define distinct TN subtypes with greater central sensitization [21,22]. Continuous pain has been linked to greater trigeminal root atrophy on high-field MRI and may reflect more advanced structural nerve injury [20]. Comorbid migraine with aura has also been associated with an increased risk of TN [21]. In our patient, the evolution from purely paroxysmal to mixed paroxysmal-continuous pain on the right side likely reflected disease progression and contributed to the decision to prioritize right-sided decompression.

Neuroinflammation should also be considered, as arachnoiditis occurs in 12.9% of patients undergoing MVD for TN, is associated with poorer outcomes, and has been correlated with elevated serum levels of cytokines such as interleukin-1β, interleukin-6, interleukin-8, and tumor necrosis factor [23]. Although our patient did not present imaging or clinical features suggestive of these secondary etiologies, they were systematically considered and excluded.

Disease burden and treatment rationale

TN imposes a substantial individual and societal burden. Up to 45% of patients restrict daily activities and more than 50% miss work, with high rates of depression (35-65%) and anxiety (19-50%), sleep disorders, and increased risks of stroke and dementia [24,25]. Pharmacological therapy remains the first-line treatment and should be adequately dosed and monitored according to European Academy of Neurology guidelines, but evidence shows that nearly two-thirds of patients continue to experience moderate to severe pain despite medication [5,12,13]. In such cases, surgical options must be considered.

Among interventional treatments, MVD is the preferred option for classical TN, providing immediate pain relief in 66-88% of patients with a low complication rate [2]. Percutaneous procedures, such as balloon compression, glycerol rhizotomy, radiofrequency thermocoagulation, and stereotactic radiosurgery, offer high initial response rates but are associated with higher rates of trigeminal sensory or motor deficits, earlier recurrence, and delayed onset of effect in the case of radiosurgery [2,12,14,23]. Long-term pain control declines over time after percutaneous procedures: pain relief decreases to approximately 54% at three years after balloon compression and from up to 85% to about 38% at five years after glycerol rhizotomy [14,23]. Gamma Knife radiosurgery for bilateral TN shows pain control rates of approximately 80% at 12 months and 65% at 36 months, but with higher recurrence rates and a delayed therapeutic effect [17,26].

Bilateral MVD in the literature: review of reported series

Several case series have evaluated the outcomes of bilateral MVD for BTN. Table 1 summarizes the main characteristics and outcomes of these series. Overall, BTN has been associated with higher treatment burden and a greater likelihood of requiring multiple procedures than unilateral TN, and outcomes tend to be less favorable when secondary etiologies or complex vascular anatomy are present. In particular, TN associated with vertebrobasilar or superior cerebellar dolichoectasia may carry a higher risk of long-term recurrence after MVD, as progressive elongation and stiffness of the dolichoectatic vessel can lead to re-displacement of the arterial loop toward the trigeminal root over time. Although our patient remains pain-free at the current follow-up, this pathophysiological background suggests that close long-term surveillance is warranted and that patients should be counselled about the potential for future recurrence in the setting of dolichoectatic neurovascular conflict.

**Table 1:** Published series of bilateral MVD for BTN Summary of selected case series reporting outcomes of bilateral microvascular decompression in patients with bilateral trigeminal neuralgia, including surgical strategy, follow-up, pain relief, and complications. TN: trigeminal neuralgia, BTN: bilateral TN, MVD: microvascular decompression, MS: multiple sclerosis, BNI: Barrow Neurological Institute

Author(s)	Year	Number of patients with BTN	BTN incidence (%)	Surgical approach for BTN	Mean follow-up	Pain relief on treated sides (%)	Complications	Findings
Pollack et al. [11]	1988	35	5.0% of 699 TN patients	Unilateral or bilateral MVD (10 patients bilateral, 22 procedures)	≈ 75 months (mean)	89% good/excellent after first MVD; 74% maintained long-term	No mortality; no major new trigeminal sensory deficits	BTN more frequent in females, familial TN, and patients with hypertension; MVD effective and safe in bilateral disease
Tacconi & Miles [16]	2000	16	Not reported	Bilateral MVD (various strategies)	≈ 14 years	Poorer outcomes; many required additional procedures	Not specified in detail	Series included idiopathic BTN and BTN associated with MS or Charcot-Marie-Tooth disease; secondary forms had less favorable results
Zhao et al. [27]	2018	13	Not reported	Bilateral MVD (9 underwent second-side MVD within 1 year)	Not clearly specified	92.3% good/excellent pain control on treated sides	Not clearly specified	Authors recommend contralateral MVD at least 3 months after first procedure when symptoms persist
Xu et al.​ [4]	2023	34	Not reported	Multiple procedures: MVD, percutaneous rhizotomies, radiosurgery for each side	5.0 ± 7.7 years	Pain significantly decreased at final follow-up; many patients required repeated procedures	Higher recurrence with glycerol and combined procedures vs MVD alone	BTN associated with high treatment burden; MVD more durable than ablative procedures, but many patients remain medication-dependent
Segura-Lozano et al. [15]	2024	15 (3 consecutive, 12 non-consecutive)	6.6% of 2,166 classical TN patients	Immediate consecutive bilateral MVD (same session) vs non-consecutive staged MVD	≥ 6 months	Significant pain relief in both groups; BNI scores comparable (p = 0.305)	Mostly transient complications; no mortality or major adverse events	Consecutive bilateral MVD shortened total surgical time vs non-consecutive MVD and was similarly safe and effective

## Conclusions

This case illustrates the diagnostic and therapeutic complexities of simultaneous BTN, an exceptionally uncommon presentation in which distinguishing true neurovascular conflict from alternative aetiologies is crucial for guiding management. Comprehensive clinical assessment, combined with high-resolution MRI, enabled a confident diagnosis by demonstrating bilateral neurovascular compression with dolichoectasia while excluding demyelinating, infectious, and systemic autoimmune causes. Staged bilateral MVD achieved immediate and complete pain relief without complications. These findings support bilateral MVD as a safe and effective option for selected patients, underscoring the importance of meticulous, imaging-guided evaluation.
